# VPOT: A Customizable Variant Prioritization Ordering Tool for Annotated Variants

**DOI:** 10.1016/j.gpb.2019.11.001

**Published:** 2019-11-22

**Authors:** Eddie Ip, Gavin Chapman, David Winlaw, Sally L. Dunwoodie, Eleni Giannoulatou

**Affiliations:** 1 Victor Chang Cardiac Research Institute, Sydney 2010, Australia; 2 St Vincent’s Clinical School, University of New South Wales, Sydney 2052, Australia; 3 Heart Centre for Children, The Children's Hospital at Westmead, Sydney 2145, Australia; 4 Sydney Medical School, University of Sydney, Sydney 2050, Australia; 5 School of Biotechnology and Biomolecular Sciences, University of New South Wales, Sydney 2033, Australia

**Keywords:** Next-generation sequencing, Pathogenicity predictions, Variant prioritization, Customizable ranking, Genomic annotation

## Abstract

**Next-generation sequencing** (NGS) technologies generate thousands to millions of genetic variants per sample. Identification of potential disease-causal variants is labor intensive as it relies on filtering using various annotation metrics and consideration of multiple pathogenicity prediction scores. We have developed VPOT (**variant prioritization** ordering tool), a python-based command line tool that allows researchers to create a single fully customizable pathogenicity ranking score from any number of annotation values, each with a user-defined weighting. The use of VPOT can be informative when analyzing entire cohorts, as variants in a cohort can be prioritized. VPOT also provides additional functions to allow variant filtering based on a candidate gene list or by affected status in a family pedigree. VPOT outperforms similar tools in terms of efficacy, flexibility, scalability, and computational performance. VPOT is freely available for public use at GitHub (https://github.com/VCCRI/VPOT/). Documentation for installation along with a user tutorial, a default parameter file, and test data are provided.

## Introduction

With the increasing use of next-generation sequencing (NGS) methods, researchers are now faced with many genetic variants, from hundreds of thousands to millions, to evaluate. Software such as ANNOVAR and VEP [1], [2] use databases that provide functional consequences, pathogenicity predictions, and population frequencies to annotate genetic variants.

There are many pathogenicity-prediction algorithms available, such as CADD, PolyPhen-2, SIFT, and MutationTaster2 [3], [4], [5], [6], but there is no single algorithm that has been universally accepted as the best. Genetic variants predicted to be deleterious by multiple methods are likely to be of greater interest in disease studies [7]. In practice, multiple pathogenicity prediction scores are utilized to increase the likelihood of identifying a disease-causing variant. Thus, to determine if a variant is likely to be disease-causal, all prediction scores are often considered together in addition to variant filtering based on other annotation metrics (such as variant frequency in control databases). This makes the prioritization of genetic variants a labor-intensive and cumbersome task.

To facilitate this process, several variant prioritization tools have been developed. However, they are either web-based (such as Variant Ranker [8]), making the analysis of whole-genome data difficult, or they do not provide an aggregated score across all annotation values (such as VaRank [9]). We have developed variant prioritization ordering tool (VPOT), a python-based command line program that creates a single aggregated pathogenicity ranking score from any number of annotation values via customizable weighting. Using this score, VPOT ranks variants, allowing researchers to prioritize variants based on all annotation data and pathogenicity-prediction outcomes.

## Methods

**Figure 1:**
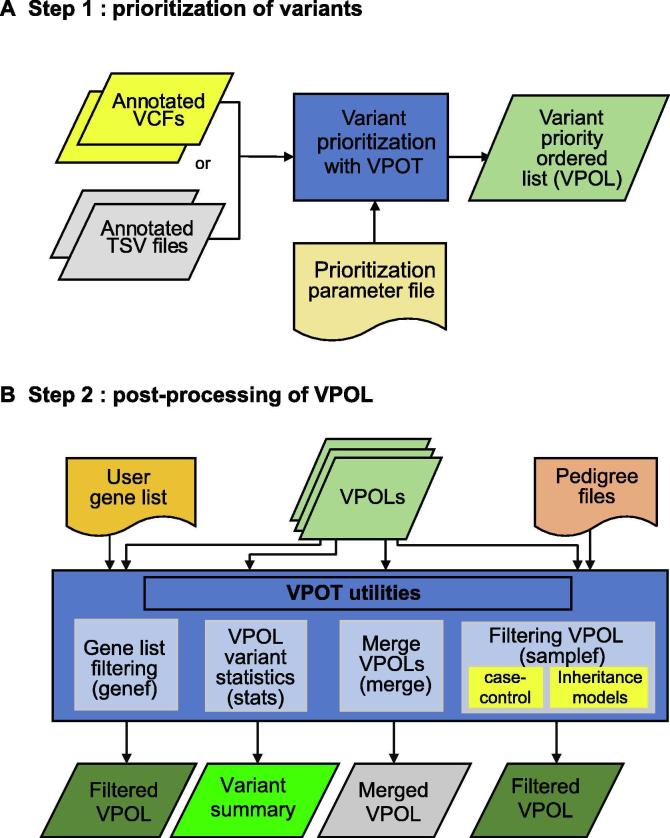
**Variant prioritization ordering tool (VPOT) workflow** **A**. Step 1: prioritization of variants. VPOT is run with annotated VCFs or TSV files and a PPF to create the VPOL. **B.** Step 2: post-processing of the variant priority ordered list (VPOL). The VPOL can be filtered based on user needs such as against a gene list for candidate gene/variants selection (genef). The VPOL can be filtered for case-control variants reporting or for inheritance models (DN/AD/AR/CH) when applied against a family trio. A pedigree format file is required and the choice of the samples filtering option (samplef). A quick variant report can be produced from the VPOL (stats), and multiple VPOL files can be combined to produce a single VPOL to allow for large cross cohort evaluation across samples (merge). VPOT, variant prioritization ordering tool; VCF, variant call format; TSV, tab-separated values; PPF, prioritization parameter file; VPOL, variant priority ordered list; DN, *de novo*; AD, autosomal dominant; AR, autosomal recessive; CH, compound heterozygous.

The VPOT workflow consists of two main steps: variant prioritization and post-processing of the variant priority ordered list (Figure 1, Figures. S1 and S2).

### Prioritization of variants

#### Creation of the prioritization parameter file (PPF)

Using ANNOVAR-annotated VCFs or tab-separated-values files (TSV, which can be annotated by any software) as input the VPOT priority function creates a prioritization parameter file (PPF) based on all the annotation elements found. The PPF will determine if the annotation fields are characters or numeric. It will list the range of values found within that field to aid customization by the user. By modifying the PPF, the user can select which annotation fields to use in the prioritization process and the weighting to apply to a specific range of values for each annotation field. Additionally, the PPF allows users to filter variants on fields attributes; for example, a population frequency threshold can be defined for Exome Aggregation Consortium (ExAC)/Genome Aggregation Database (gnomAD) [10]. A PPF only needs to be set up once as it can be applied repeatedly to prioritize variants in different samples if the annotation fields used within the PPF are available. While utilization of new prediction annotations would require modification of the PPF, VPOT will still run successfully without PPF modification, but it would utilize only the annotations indicated in the PPF.

VPOT is designed to allow the user to customize their prioritization process based on annotations relevant to the disease of study. However, we also provide a default PPF with a list of recommended annotations based on our experience with one complex disease, congenital heart disease [11], [12]. The default PPF eliminates variants with a minor allele frequency higher than 0.1% with respect to control databases (ExAC, gnomAD, and 1000 Genomes Project), low quality variants (with coverage less than 8 times or 25% allelic balance), and synonymous variants. The weighting criteria for each *in silico* predictor used in the default PPF are set to identify pathogenic variants based on pathogenicity threshold recommended by the individual algorithms or informed by the literature (*e.g.*, CADD, PolyPhen-2, MutationTaster2, LRT, MCAP, GERP++, MetaSVM [13], [14], [15], [16]). The default PPF also weighs the most disruptive variants such as stop-gain, frameshift indels, and splicing variants highly.

#### Creation of the variant priority ordered list (VPOL)

Annotated VCFs/TSV files and a PPF are passed as input to VPOT to perform the prioritization function on all variants. Using the PPF-customized weights, each variant is scored by aggregating all the user-defined values. This is done by calculating the sum of all encoded weights for each variant. A normalized score is also calculated by dividing by the maximum score found across all variants. All variants by default are returned and ordered in the output, which we call the variant priority ordered list (VPOL). Variants with low score (*e.g.*, synonymous variants) can be removed at this stage by providing a cutoff within the PPF so that only variants with scores greater or equal to the cutoff are included in the VPOL.

For each variant, VPOT performs quality control checks on each sample’s genotype based on coverage (number of reads at variant position) and allele balance (percentage of alternate allele reads at variant position). The user, via the PPF, can customize the quality control check thresholds. If the sample genotype call fails these quality control checks, then it is marked in the VPOL. For each variant line in the VPOL each sample’s genotype is denoted as, “0” for reference, “1” for heterozygous, “2” for homozygous alternate, or “.” for quality control failure. This prioritization step can be easily performed in parallel across many samples or repeated for new samples by using the same PPF as part of the input.

### Post-processing of the VPOL

VPOT provides several post-prioritization options to explore the VPOL (Figure 1). A summary statistics option (stats) generates a quick and simple variant report for the supplied VPOL highlighting the number of scored variants, and a list of genes that score in the top 75th percentile (default) of variants found for each sample in the VPOL. VPOT allows researchers to apply a user-defined candidate gene list to filter any VPOL using the gene filtering option (genef).

VPOT can filter variants in the VPOL based on inheritance or absence from controls via the use of the sample filtering option (samplef). This option utilizes a ped (pedigree) format file. The sample filtering option can filter variants based on their case-control status by extracting variants that exist in case samples and not in control samples of a large cohort. The VPOT samplef option can also filter variants based on different Mendelian inheritance models. A complete family trio, defined by the presence of parents and proband, is required for this option. The *de novo* (DN) model identifies variants that only exist in the proband and not in any of the parents. The autosomal dominant (AD) model identifies variants that exist in both the proband and affected parent but not in the unaffected parent. The autosomal recessive (AR) model identifies variants that are homozygous for the alternative allele in the proband and heterozygous in both parents. The compound heterozygous (CH) model provides a filter that returns heterozygous variants in genes that have both proband-paternal and proband-maternal specific variants.

For large cohort studies, it is recommended to run multiple VPOT processes for small subsets of samples in parallel to reduce computational time. To facilitate the ability to view all the samples in a single VPOL file, VPOT has a merge option (merge) to consolidate multiple numbers of VPOL files back to one VPOL.

## Results

### Application of VPOT to disease cohorts

**Figure 2:**
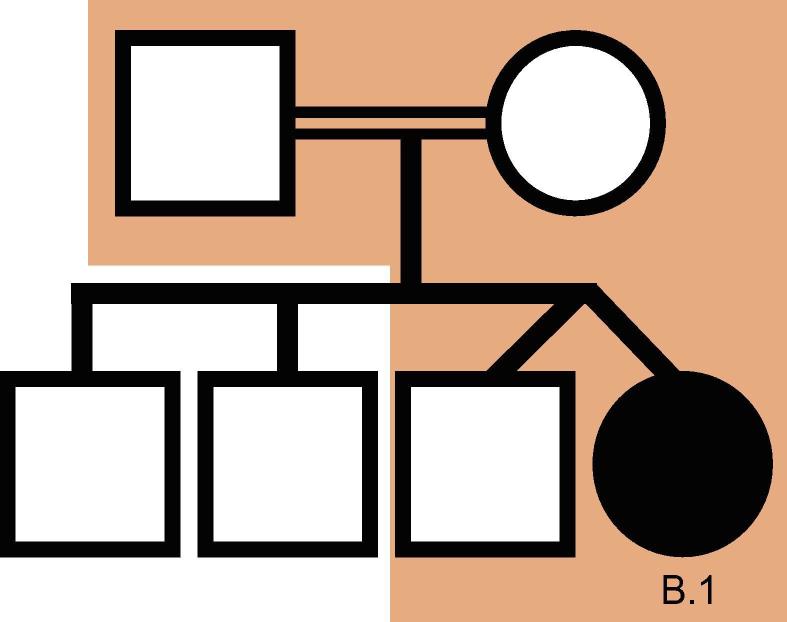
***HAAO* CHD family pedigree**. Family B from [17] is a consanguineous family, with proband sample B.1 having CHD and other extra-cardiac phenotypes and all other siblings being unaffected. Samples within the shaded region of the pedigree have undergone whole genome sequencing. CHD, congenital heart disease.

We used VPOT to identify potentially pathogenic gene variants in a family with a proband that had multiple congenital malformations (family B in Shi, et al. [17]). The family was subjected to whole-genome sequencing (WGS) and over 7.7 million variants identified. Following filtering and prioritization by VPOT using the default PPF the number of candidate variants decreased to 587. Based on the family pedigree which shows that the parents were consanguineous, we used VPOT’s inheritance model filtering (within samplef option) to refine the number of candidate variants based on an autosomal recessive inheritance model (AR) (Figure 2). After application of inheritance model filtering, 14 variants remained with a *HAAO* homozygous variant ranked first, consistent with the reported genetic cause in this family (Table 1 and Table S1) [17]. The identification of the *HAAO* variant demonstrates the ability of VPOT to facilitate monogenic disease variant discovery in a systematic way.

**Table 1:** **Top ten variants for family B following autosomal recessive inheritance model filtering (Samplef – AR)**.

**Priority score**	**Gene**	**DNA variant**	**Exonic function**	**gnomAD**	**LRT**	**MutationTaster2**	**PolyPhen-2 HVAR**	**CADD**	**MetaSVM**	**GERP++**
67	*HAAO*	c.558G > A	Stop-gain	4.07E−06	D	Adc	NA	39	NA	5.26
57	*CNOT2*	c.1621_1622insAAAAA	FS-I	NA	NA	NA	NA	NA	NA	NA
30	*SLC52A2*	c.916G > A	NS-SNV	5.14E−05	D	Dc	Dp	28.3	Dm	4.69
27	*MAPK15*	c.419C > T	NS-SNV	0.000384	D	Dc	Dp	32	T	4.02
24	*CLTB*	c.457A > G	NS-SNV	0.000134	D	Dc	P	21.9	T	4.16
23	*SMYD5*	c.625C > A	NS-SNV	NA	D	Dc	Dp	28.4	T	3.76
23	*GAD1*	c.184C > T	NS-SNV	9.02E−05	N	Dc	P	26.4	Dm	4.66
21	*DAB2IP*	c.2186 T > A	NS-SNV	6.23E−05	N	Dc	Dp	18.1	T	4.69
18	*PSME4*	c.2074C > A	NS-SNV	NA	D	Dc	P	20.3	T	4.42
18	*WNT10A*	c.685C > G	NS-SNV	NA	D	Dc	P	25.5	T	3.57

*Note*: Detail of top ten variants for Family B [17]. VPOT prioritization was performed using the default PPF supplied within GitHub (https://github.com/VCCRI/VPOT/). LRT values – D (deleterious, when LRT value = 0.000), N (neutral). MutationTaster2 values – Adc (disease-causing automatic, when probability value from Bayes classifier used is >0.5 and variant is marked as probable-pathogenic or pathogenic in ClinVar), Dc (disease-causing, when probability value from Bayes classifier used is >0.5). PolyPhen-2 HVAR values – Dp (probably damaging, when naïve Bayes posterior probability of damaging’s estimate of false positive rate is ≤10%), P (possibly damaging, when naïve Bayes posterior probability of damaging’s estimate of false positive rate is ≤20%). MetaSVM values – Dm (deleterious, when value >0), T (tolerated). See Table S1 for full scoring details with all predictors’ values. FS-I, Frameshift-insertion; NS-SNV, non-synonymous single nucleotide variant; NA, not applicable; gnomAD, genome aggregation database; LRT, likelihood ratio test; CADD, combined annotation dependent depletion; GERP, genomic evolutionary rate profiling.

VPOT has been successfully used to prioritize variants in a congenital heart disease (CHD) cohort of 30 families that were whole-exome sequenced [11], with the disease-causing variants in the three solved families ranked within the top 2% of all variants found. In another cohort of 97 CHD families that underwent WGS [12], clinically actionable variants were identified in 28 families, and VPOT ranked the majority of these variants within the top 1% of all variants found. Only two variants were not ranked within the top 1% of variants due to large disagreement in pathogenicity prediction between different methods. We have provided the PPF file used for the prioritization of variants in these studies as a default PPF for the study of complex diseases like CHD.

### Comparison with existing variant prioritization tools

VPOT’s approach to variant prioritization is to aggregate pathogenicity predictor scores since no single pathogenicity predictor score has been shown to predict pathogenic mutations reliably. Other packages have utilized this same approach, and we identified two for evaluation comparison that are most similar to VPOT, Variant Ranker [8] – a web-based tool, and VaRank [9] – a command line program. Both Variant Ranker and VaRank create a ranking value for variants based on a set of user-defined scores for pathogenicity predictors like VPOT.

We compared the overall features and functionality between the tools (Table 2). Both VPOT and VaRank have no restriction on the input file size, which is important for the analysis of variants resulting from whole genome sequencing. Annotation is controlled by the user for both VPOT and VaRank, although it is a separate process for VPOT and part of the tool for VaRank. This provides greater flexibility for the user to adopt newer releases of the human reference genome, and novel pathogenicity predictors, such as for splicing and non-coding genetic variants. For Variant Ranker, the variant annotation process is embedded within its workflow and cannot be modified by the user. All three tools rank variants based on the scores of multiple pathogenicity prediction methods. However the number of predictors vary, with the lowest seen in VaRank that uses only three fixed pathogenicity prediction tools (phastCons [18], SIFT, and PolyPhen-2), then Variant Ranker that uses seven fixed tools (PolyPhen-2, SIFT, LRT, MutationTaster2, MutationAssessor, RadialSVM, and FATHMM [16], [19], [20]), and finally VPOT where the number is limited only by the predictors included in the annotation. Accounting for differences in the genetic architectures of different diseases, VPOT allows expert users to apply their specialized knowledge of disease to stratify results from *in silico* predictors. The user can select higher weighting for specific predictors to enhance the accuracy for the disease or study design in question. VPOT also allows fine-tuning of variant ranking as the user can define any number of scoring intervals for an annotation category. This allows the user to define different pathogenicity thresholds instead of a binary non-damaging/damaging scenario. Finally, both VPOT and VaRank are local machine tools, so there is no security concern with sensitive study data being stored in the cloud.

**Table 2:** **Feature comparison of VPOT with similar variant prioritization tools**.

**Feature**	**VPOT**	**VaRank (v1.4.2)**	**Variant Ranker**
Process location	Local	Local	Web

Input format	VCF (gz)/TXT (multiple files)	VCF (gz) (multiple files)	VCF/TXT (single file)

File size limit	No limit	No limit	500 MB

Annotation	ANNOVAR (freeware), performed by user prior to using tool	Alamut (commercial tool)/SnpEff (freeware), performed by tool	ANNOVAR (freeware), performed by tool

Reference genome	No restriction	No restriction	Hg19

Annotation resources that can be applied to VCF	User-defined	User-defined	Defined by tool

Pathogenicity prediction tools supported	Based on user-defined annotations (no limit)	phastCons, SIFT, PolyPhen-2	PolyPhen-2, SIFT, LRT, MutationTaster, MutationAssessor, RadialSVM, FATHMM

Disease/inheritance model	DN/AD/AR/CH	DN/AD/AR/CH	AD/AR/XR

Quality control check	Total coverage depth, allele balance	NA	Total coverage depth, variant allele coverage depth, allele balance

Score weighting range	User-defined	User-defined	0–1

Number of scoring intervals for each annotation category	User-defined	NA	Defined by tool

Output format	TXT – Local	TSV – Local	TXT – Web

*Note*: DN, *De novo*; AD, autosomal dominant; AR, autosomal recessive; CH, compound heterozygous; XR, X-linked-recessive.

We evaluated VPOT, VaRank, and Variant Ranker by prioritizing variants from an exome sequencing dataset on idiopathic hemolytic anemia (MIM:266200) [21] used previously by Variant Ranker to demonstrate its effectiveness [8]. Following as close as possible the default variant scoring criteria of Variant Ranker, VPOT also ranked the most likely causative gene *PKLR* in the fourth position like Variant Ranker. We were not able to replicate the same scoring parameters as Variant Ranker using VaRank due to the limited number of pathogenicity predictors scoring options. With VaRank, using its default scoring parameters the *PKLR* variant was ranked in 199th position with an annotation impact value of “Moderate”. Both VaRank and Variant Ranker provide CADD Phred score annotation but do not include it in their final ranking. CADD score is a commonly used pathogenicity predictor, and a minimum score of 20 has been used as a lower threshold for variants considered to be possibly pathogenic [14]. Utilizing the flexibility of VPOT we added CADD into our annotation and PPF with a weighting for CADD Phred score above 20. Under these new ranking criteria, the *PKLR* variant was ranked first by VPOT. This demonstrates the benefit of VPOT’s customizability to allow the users to refine and tune the variant prioritization process.

**Figure 3:**
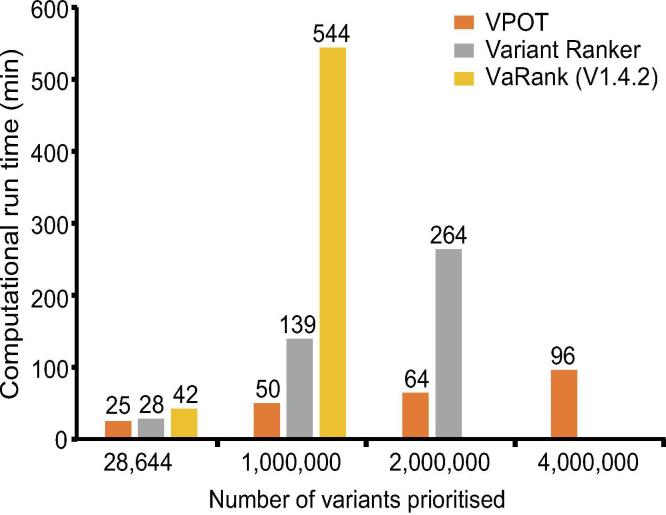
**Comparison of computational performance of VPOT with similar variant prioritization tools**. Prioritization computational time measurements for VPOT, VaRank, Variant Ranker against number of variants. Processing time limitation (48 h) was exceeded by VaRank when attempting ≥2 million variants. File size limitation exceeded for Variant Ranker when attempting >2 million variants. More information on the settings and parameters used is provided in Table S2.

Finally, we compared the computational performance of the three tools when ranking files with different number of variants (Figure 3 and Table S2). The processing time for VPOT and VaRank includes the annotation of the input VCF (to emulate the Variant Ranker processing which includes its annotation). VPOT was consistently faster than both VaRank and Variant Ranker, and as the number of variants increased the time difference between VPOT and the others were magnified. Additionally, VPOT was the only tool able to complete variant prioritization task for samples containing up to four million variants. In comparing the amount of central processing unit memory usage for the local machine tools, VPOT required a significantly smaller amount of memory to perform the prioritization tasks compared to VaRank.

## Conclusion

VPOT provides a convenient way to prioritize genetic variants in disease sequencing studies. It is fully customizable, allowing researchers to filter on any annotation metrics and set weights for pathogenicity predictions that reflect their specific disease-variant hypothesis in question. The use of VPOT can be especially informative when analyzing sequencing cohorts containing many families, as the prioritization of variants can allow researchers to identify most likely disease-causal candidate variants quickly across all families.

VPOT is highly scalable for large genome analysis. Whole-genome sequencing generates very large variant files, and there are now increasing requirements for prioritization of non-coding variants that make up ∼98% of the genome. As larger sequencing studies are performed, VPOT will further prove to be an extremely valuable tool.

## Availability

VPOT is freely available for public use at GitHub (https://github.com/VCCRI/VPOT/). Documentation for installation along with a user tutorial, default parameter file, and test data are provided. Additional datasets analyzed in the current study are available upon request from the corresponding author.

## Authors’ contributions

EI developed the application tool, performed the analyses and drafted the paper. GC, DW and SLD participated in the design of the tool. EG participated in the design and helped to draft the manuscript. All authors read and approved the final manuscript.

## Competing interests

The authors have declared no competing interests.
