# Supplementary figures and images for: VPOT: A Customizable Variant Prioritization Ordering Tool for Annotated Variants
Source: Genomics Proteomics Bioinformatics. 2019 Nov 22;17(5):540–5. doi: 10.1016/j.gpb.2019.11.001 (PMC7056850; doi:10.1016/j.gpb.2019.11.001)

## Step 1 : prioritisation of variants

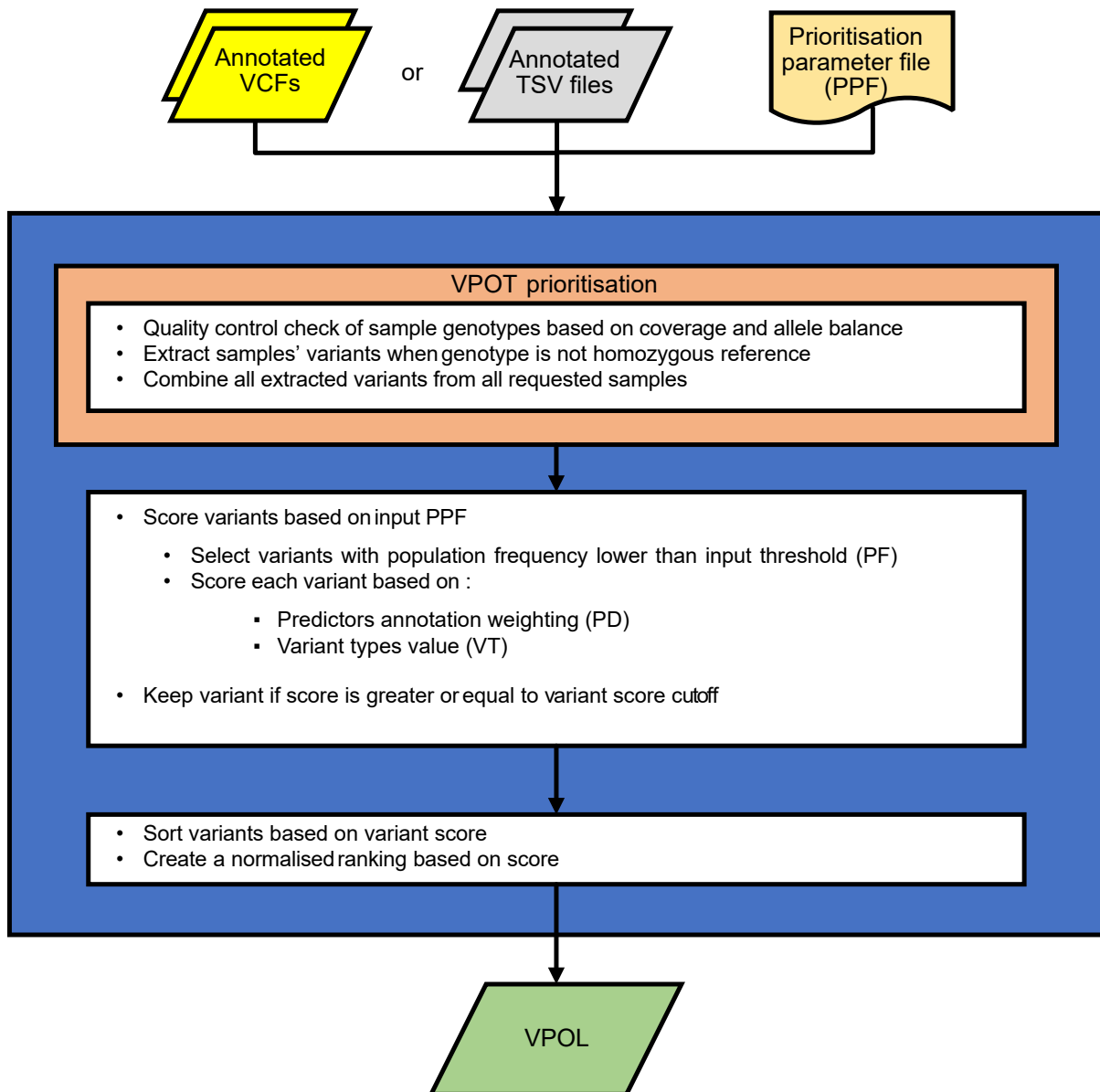

Supplement: Supplementary Fig. S1 [file mmc1.pdf]
