# Supplementary material for: VPOT: A Customizable Variant Prioritization Ordering Tool for Annotated Variants
Source: Genomics Proteomics Bioinformatics. 2019 Nov 22;17(5):540–5. doi: 10.1016/j.gpb.2019.11.001 (PMC7056850; doi:10.1016/j.gpb.2019.11.001)
Supplement: Supplementary Fig. S2 [file mmc2.pdf]

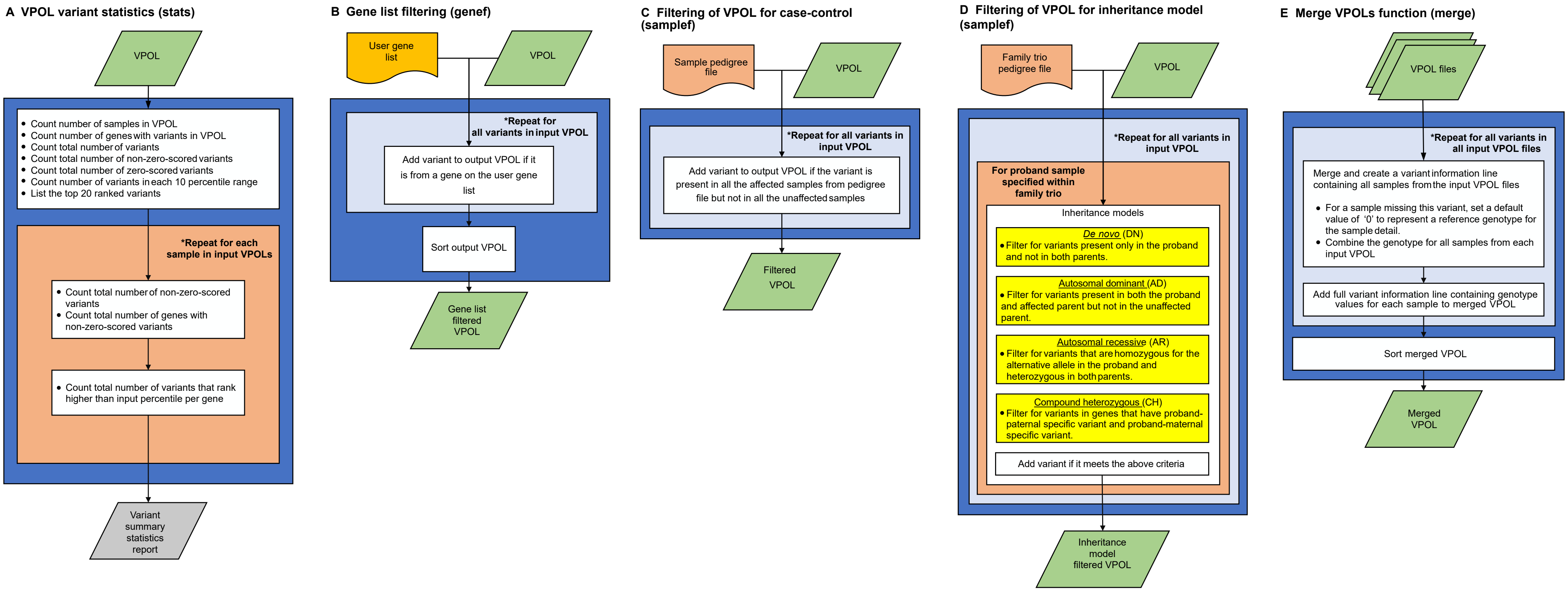
A VPOL variant statistics (stats)
VPOL
Count number of samples in VPOL
Count number of genes with variants in VPOL
Count total number of variants
Count total number of non-zero-scored variants
Count total number of zero-scored variants
Count number of variants in each 10 percentile range
List the top 20 ranked variants
*Repeat for each sample in input VPOLs
Count total number of non-zero-scored variants
Count total number of genes with non-zero-scored variants
Count total number of variants that rank higher than input percentile per gene
Variant summary statistics report
B Gene list filtering (genef)
User gene list
VPOL
*Repeat for all variants in input VPOL
Add variant to output VPOL if it is from a gene on the user gene list
Sort output VPOL
Gene list filtered VPOL
C Filtering of VPOL for case-control (samplef)
Sample pedigree file
VPOL
*Repeat for all variants in input VPOL
Add variant to output VPOL if the variant is present in all the affected samples from pedigree file but not in all the unaffected samples
Filtered VPOL
D Filtering of VPOL for inheritance model (samplef)
Family trio pedigree file
VPOL
*Repeat for all variants in input VPOL
For proband sample specified within family trio
Inheritance models
De novo (DN)
Filter for variants present only in the proband and not in both parents.
Autosomal dominant (AD)
Filter for variants present in both the proband and affected parent but not in the unaffected parent.
Autosomal recessive (AR)
Filter for variants that are homozygous for the alternative allele in the proband and heterozygous in both parents.
Compound heterozygous (CH)
Filter for variants in genes that have proband-paternal specific variant and proband-maternal specific variant.
Add variant if it meets the above criteria
Inheritance model filtered VPOL
E Merge VPOLs function (merge)
VPOL files
*Repeat for all variants in all input VPOL files
Merge and create a variant information line containing all samples from the input VPOL files
For a sample missing this variant, set a default value of '0' to represent a reference genotype for the sample detail.
Combine the genotype for all samples from each input VPOL
Add full variant information line containing genotype values for each sample to merged VPOL
Sort merged VPOL
Merged VPOL
