# Supplementary material for: VPOT: A Customizable Variant Prioritization Ordering Tool for Annotated Variants
Source: Genomics Proteomics Bioinformatics. 2019 Nov 22;17(5):540–5. doi: 10.1016/j.gpb.2019.11.001 (PMC7056850; doi:10.1016/j.gpb.2019.11.001)
Supplement: Supplementary Table S1 [file mmc3.docx]

**Table S1 Top ten variants for family B following autosomal recessive inheritance model filtering (Samplef – AR)**

| **Ranking** | **Priority score** | **Gene** | **DNA variant** | **Exonic function** | **gnomAD** | **ExAC** | **1000g** | **LRT** | **MutationTaster2** | **SIFT** | **PolyPhen-2 HDIV** | **PolyPhen-2 HVAR** | **CADD** | **MCAP** | **GERP++** | **phyloP20way mammalian** | **MutationAssessor** | **FATHMM** | **PROVEAN** | **Fathmm MKL** | **MetaSVM** |
| --- | --- | --- | --- | --- | --- | --- | --- | --- | --- | --- | --- | --- | --- | --- | --- | --- | --- | --- | --- | --- | --- |
| 0.97 | 67 | *HAAO* | c.558G>A | stopgain | 4.07E–06 | NA | NA | D | Adc | NA | NA | NA | 39 | NA | 5.26 | 0.843 | NA | NA | NA | Df | NA |
| 0.83 | 57 | *CNOT2* | c.1621_1622insAAAAA | FS-I | NA | NA | NA | NA | NA | NA | NA | NA | NA | NA | NA | NA | NA | NA | NA | NA | NA |
| 0.43 | 30 | *SLC52A2* | c.916G>A | NS-SNV | 5.14E–05 | 3.36E–05 | NA | D | Dc | Ds | pD | Dp | 28.3 | 0.641 | 4.69 | 0.953 | Medium | Tolerated | Dv | Df | Dm |
| 0.39 | 27 | *MAPK15* | c.419C>T | NS-SNV | 0.000384 | 0.0005 | 0.001 | D | Dc | Ds | pD | Dp | 32 | 0.355 | 4.02 | 0.788 | Medium | Tolerated | Dv | Df | Tolerated |
| 0.35 | 24 | *CLTB* | c.457A>G | NS-SNV | 0.000134 | 0.0002 | 0.0002 | D | Dc | Ds | Pd | P | 21.9 | 0.026 | 4.16 | 0.906 | Medium | NA | Dv | Df | Tolerated |
| 0.33 | 23 | *SMYD5* | c.625C>A | NS-SNV | NA | NA | NA | D | Dc | Ds | pD | Dp | 28.4 | 0.110 | 3.76 | 0.852 | Medium | Tolerated | Neutral | Df | Tolerated |
| 0.33 | 23 | *GAD1* | c.184C>T | NS-SNV | 9.02E–05 | 0.0001 | NA | Neutral | Dc | Ds | pD | P | 26.4 | 0.277 | 4.66 | 0.841 | Medium | Tolerated | Neutral | Df | Dm |
| 0.3 | 21 | *DAB2IP* | c.2186T>A | NS-SNV | 6.23E–05 | 8.22E–05 | NA | Neutral | Dc | Tolerated | pD | Dp | 18.14 | 0.025 | 4.69 | 1.058 | Medium | Tolerated | Neutral | Df | Tolerated |
| 0.26 | 18 | *PSME4* | c.2074C>A | NS-SNV | NA | NA | NA | D | Dc | Tolerated | pD | P | 20.3 | 0.019 | 4.42 | 1.048 | Low | Tolerated | Neutral | Df | Tolerated |
| 0.26 | 18 | *WNT10A* | c.685C>G | NS-SNV | NA | NA | NA | D | Dc | Ds | Pd | P | 25.5 | 0.061 | 3.57 | 0.852 | Low | Tolerated | Neutral | Df | Tolerated |

*Note*: Detail of top ten variants for Family B [17]. VPOT prioritisation performed using the default PPF supplied within GitHub (https://github.com/VCCRI/VPOT/). LRT values – D, when value = 0.000. Mutation Taster values – Adc (disease-causing Automatic), when probability value from Bayes classifier used is > 0.5 and variant marked as probable-pathogenic or pathogenic in ClinVar; Dc (Disease-causing), when probability value from Bayes classifier used is > 0.5. PolyPhen-2 HVAR values – Dp (probably damaging), when naïve Bayes posterior probability of damaging’s estimate of false positive rate is <= 10%; P (possibly damaging), when naïve Bayes posterior probability of damaging’s estimate of false positive rate is <= 20%. PolyPhen-2 HDIV values – pD (probably damaging), when naïve Bayes posterior probability of damaging’s estimate of false positive rate is <= 5%; Pd (possibly damaging), when naïve Bayes posterior probability of damaging’s estimate of false positive rate is <= 10%. MetaSVM values – Dm (Deleterious), when value > 0. SIFT values – Ds (Deleterious), when value < 0.05. CADD - > 10 for pathogenicity threshold. MCAP - > 0.025 pathogenicity threshold. GERP++ - > 4 for pathogenicity threshold. PhyloP20way_mammalian - > 0.9 for pathogenicity threshold. MutationAssessor - Medium, when functional impact score is > 1.9 but <= 3.5; Low, when functional impact score is > 0.8 but <= 1.9. Fathmm MKL – Df (Deleterious) - when value > 0.5. PROVEAN – Dv (Deleterious) - when score is <= -2.5. FS-I, Frameshift-insertion; NS-SNV, non-synonymous single nucleotide variant; NA, not applicable; ExAC, exome aggregation consortium; gnomAD, genome aggregation database.
