# Supplementary material for: VPOT: A Customizable Variant Prioritization Ordering Tool for Annotated Variants
Source: Genomics Proteomics Bioinformatics. 2019 Nov 22;17(5):540–5. doi: 10.1016/j.gpb.2019.11.001 (PMC7056850; doi:10.1016/j.gpb.2019.11.001)
Supplement: Supplementary Table S2 [file mmc4.docx]

**Table S2 Comparison of computational performance of VPOT with similar variant prioritisation tools**

|  | **VPOT (VCF Annotation+VPOT)** | **VaRank(v1.4.2)** | **Variant Ranker (Upload+Processing+Download)** |
| --- | --- | --- | --- |
|  |  |  |  |
| Idiopathic hemolytic anemia VCF (28,644 variants) | (24mins+30secs) = 25mins | 42mins | (3mins+25mins+2secs) = 28mins |
| 1 million variants | (40mins+10mins) = 50 mins | 544mins | (32mins+1hr46mins+1min) = 139mins |
| 2 million variants | (47mins+17mins) = 64mins | NA | (1hr3mins+3hrs20mins+1min) =264 mins |
| 4 million variants | (1hr15mins+21mins) = 96mins | NA | NA |

*Note*: VPOT computational performance times were derived from combining VCF Annotation time with Annovar (ran on 1 CPU (Intel Xeon E5-2670 @ 2.60 GHz) with 6GB memory) and VPOT prioritization (ran on 1 CPU (Intel Xeon E5-2630 @ 2.60 GHz) with – 4GB memory). VaRank prioritization utilized the freeware tool SnpEff for annotation to match the freeware tool Annovar used by VPOT and Variant Ranker. VaRank prioritization for Idiopathic hemolytic anemia VCF ran on 1 CPU (Intel Xeon E5-2670 @ 2.60 GHz) with 6GB memory. VaRank prioritization for 1 million variants VCF ran on 1 CPU (Intel Xeon E5-2670 @ 2.60 GHz) with 12GB memory. Not applicable results for VaRank tests represent inability to complete prioritization due to processing time limitation (48hrs) exceeded. Not applicable result for Variant Ranker test represents inability to complete prioritization due to file size limitation on input upload. CPU, central processing unit.
